# Complete chloroplast genome of an endangered endemic tree, *handeliodendron bodinieri* (levl.) rehder (sapindaceae) from karst forests of southwest China

**DOI:** 10.1080/23802359.2019.1671251

**Published:** 2019-09-27

**Authors:** Xinmin Tian, Xueli Li, Haoyu Miao, Chunlin Xue, Bolun Wang, Yu Guo, Gucheng Zhang

**Affiliations:** a College of Life science and Technology, Xinjiang University, Urumqi, China;; b Hainan Geological Survey Institute, Haikou, China

**Keywords:** *Handeliodendron bodinieri*, chloroplast genome, conservation

## Abstract

*Handeliodendron bodinieri* (Sapindaceae) is an endangered monotypic species endemic to the karst forests in southwest China whose populations are now fragmented and the total number of individuals evidently decreased. In this research, we characterized the chloroplast (cp) genome of *H. bodinieri* using genome skimming. The whole cp genome was 155,291 bp long and comprised 137 genes, including 8 unique rRNAs, 40 tRNAs, and 89 protein-coding genes. The overall guanine-cytosine content of *H. bodinieri* cp genome was 37.8%. The phylogenetic analysis suggested that *H. bodinieri* is closely related to the genus *Mangifera*. This study will be useful for future studies on conservation genetics of this economically important endemic plant.

*Handeliodendron bodinieri* (Levl.) Rehder, an endemic, monotypic species listed in the Chinese National Protected Plants List, mainly occurs in the karst forests of southwest China (Wu 2007). It is of high economic value in the oil industry, furniture industry, and landscaping. However, wild populations of *H. bodinieri* have been decreasing and the total number of individuals decreased evidently as a consequence of habitat destruction. Thus, effective conservation methods are necessary to protect this endangered species. Although some genetic studies were focused on *H. bodinieri* (Wang et al. 2008; He et al. 2012), a credible characterization of its cp genome is still lacking. Therefore, the characterization of the complete cp genome of *H. bodinieri* is necessary in order to develop a reasonable conservation strategy for this endangered tree species. In this study, we sequenced, assembled, and annotated the complete cp genome of *H. bodinieri* using the Illumina pair-end sequencing and genome skimming.

Fresh silica-dried leaves of *H. bodinieri* were collected from Danzhai, Guizhou, China (N 26°13′59.16″, E 107°55′8.04″). Voucher specimens were deposited in the Herbarium of the College of Life Science and Technology, Xinjiang University, Urumqi, Xinjiang (Accession No: TXM2018216). Total genomic DNA was extracted using the traditional cetyl trimethylammonium bromide (CTAB) method (Doyle and Doyle 1987). Sequencing was conducted on the Illumina Hiseq Platform (Illumina, San Diego, CA, USA). In total, we obtained 5.0 GB of raw data and approximately 20 million high-quality clean reads. The cp genome was assembled using the Velvet software (Zerbino and Birney 2008). Annotation analysis was performed with Plastome Annotator (Plann) (Huang and Cronk 2015). Afterwards, we analyzed and corrected the annotations with Geneious software (Kearse et al. 2012). We generated a physical map of the genome using Organellar Genome DRAW (OGDRAW) (Lohse et al. 2013). A maximum likelihood (ML) tree was constructed with RaxML software v. 8 (Stamatakis 2014) using cp genomes of 19 species belonging to nine angiosperm families. Finally, the complete cp sequence and genome annotations were submitted to GenBank with an accession numbers of MN367918 for *H. bodinieri*.

　　The cp genome of *H. bodinieri* had a typical quadripartite structure with a length of 155,291 bp, which contained inverted repeats (IRs) of 25,674 bp separated by a large single copy region (LSC) of 85,090 bp and a small single copy region (SSC) of 18,853 bp. The cpDNA contained 137 genes comprising 89 protein-coding genes, 40 tRNA genes, 8 rRNA genes. Among the annotated genes, 14 of them contain one intron (*atp*F, *ndh*A, *ndh*B, *pet*D, *pet*B, *rpl*2, *rpl*16, *rps*16, *rpo*C1, *trn*A-UGC, *trn*I-GAU, *trn*K-UUU, *trn*L-UAA and *trn*V-UAC),and three genes (*clp*P, *rps*12 and *ycf*3) contain two introns. The overall guanine-cytosine (GC) content of the plastome is 37.8%. Additionally, phylogenetic analysis of 19 plastid genomes showed that the cp genome of *H. bodinieri* is closely related to the genus *Mangifera* (Figure 1). The obtained genetic data can be important resource for developing future conservation and management strategies for this economically important tree.

**Figure 1.**
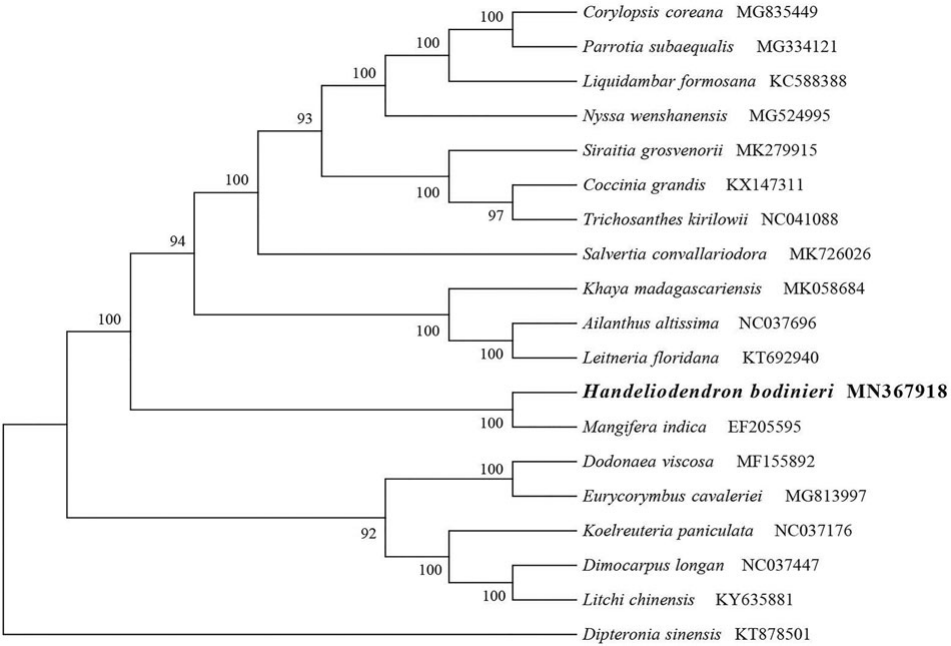
The phylogenetic tree based on the 19 complete chloroplast genome sequences. Accession numbers: *Handeliodendron bodinieri* (MN367918), *Corylopsis coreana* (MG835449), *Parrotia subaequalis* (MG334121), *Liquidambar formosana* (KC588388), *Nyssa wenshanensis* (MG524995), *Siraitic grosvenorii* (MK279915), *Coccinia grandis* (KX147311), *Trichosanthes kirilowii* (NC041088), *Salvertia convalluriodoru* (MK726026), *Khaya madagascariensis* (MK058684), *Ailanthus altissima* (NC037696), *Leitneria floridana* (KT692940), *Mangifera indica* (EF205595), *Dodonaea viscosa* (MF155892), *Eurycorymbus cavaleriei* (MG813997), *Koelreuteria paniculata* (NC037176), *Dimocarpus longan* (NC037447), *Litchi chinensis* (KY63588 l), *Dipteronia sinensis* (KT878501).
